# Tumor associated macrophages in breast cancer progression: implications and clinical relevance

**DOI:** 10.3389/fimmu.2024.1441820

**Published:** 2024-07-09

**Authors:** Maria Stavrou, Anastasia Constantinidou

**Affiliations:** ^1^ Department of Translational Research and Precision Medicine, Cyprus Cancer Research Institute (CCRI), Nicosia, Cyprus; ^2^ Medical School, University of Cyprus, Nicosia, Cyprus; ^3^ Department of Medical Oncology, Bank of Cyprus Oncology Centre, Strovolos, Cyprus

**Keywords:** breast cancer, tumor associated macrophages, surface markers, prognosis, metastasis

## Abstract

Macrophages represent an immune cell population characterized by high plasticity and a range of properties and functions. Their activation status and specific phenotype are highly associated with their localization and the environmental cues they receive. The roles of macrophages in cancer development are diverse. Despite their antitumor effects at early stages of the disease, their presence in the tumor microenvironment (TME) has been linked to tumor promotion upon disease establishment. Tumor associated macrophages (TAMs) are key components of breast cancer TME and they have been associated with poor clinical outcomes. High TAM densities were found to correlate with tumor progression, increased metastatic potential and poor prognosis. Interestingly, considerably higher levels of TAMs were found in patients with triple negative breast cancer (TNBC)—the most aggressive type of breast cancer—compared to other types. The present review summarizes recent findings regarding the distinct TAM subsets in the TME and TAM involvement in breast cancer progression and metastasis. It highlights the constant interplay between TAMs and breast cancer cells and its major contribution to the progression of the disease, including such aspects as, polarization of macrophages toward a tumor promoting phenotype, induction of epithelial to mesenchymal transition (EMT) in cancer cells and enhancement of cancer stem cell properties. Further, we discuss the clinical relevance of these findings, focusing on how a better delineation of TAM involvement in breast cancer metastasis will facilitate the selection of more efficient treatment options.

## 1 Introduction

Breast cancer (BC) is among the most frequently diagnosed types of cancer worldwide and the second leading cause of cancer related mortality in women (1–3). It can be subdivided into different types based on the expression of estrogen (ER) and progesterone (PR) hormone receptors, HER2 expression and Ki67 (4, 5). Triple negative breast cancer (TNBC), ER^-^/PR^-^/HER2^-^, accounts for 10–20% of BC and it is characterized by high aggressiveness and poor prognosis owing to the lack of targeted therapeutic strategies (6, 7). A substantial amount of evidence has supported the involvement of tumor associated macrophages (TAMs) in cancer progression and metastasis in various types of cancer, including BC (8–10). In fact, TAMs represent the dominant immune cell population of BC tumor microenvironment (TME) and they have been correlated with poor prognosis and increased metastatic potential. Distinct TAM subsets can differentially affect disease progression, and this is highly dictated by their specific phenotype, and their spatial and temporal distribution (11, 12). TAM subsets in BC TME have also been utilized as predictive tools of clinical outcomes. A thorough characterization of the TAM signature of individual BC patients could facilitate the design of personalized and more efficient treatment strategies. It could also enable a more accurate prediction of patients’ response to treatment. In this review, we summarize subtypes of TAMs commonly encountered in the TME of BC, highlighting the heterogeneity and diversity of these cells. In addition, we present recent findings concerning TAM involvement in BC progression and metastasis, with particular attention to the constant crosstalk between TAMs and cancer cells and its central role in fueling and maintaining disease progression.

## 2 Distinct TAM subtypes in breast cancer progression and prognosis

In an oversimplified manner, macrophages in the TME were previously distinguished into the pro-inflammatory M1 type-linked to antitumor functions and the anti-inflammatory M2 type-endowed with tumor promoting capabilities (13). This M1/M2 distinction represents the two extremes, and macrophages of intermediate states are also present in the TME. More recently, the terms M1-like and M2-like macrophages were introduced to refer to anti- and pro-tumor macrophages respectively (14, 15). Still, there is a grey zone in this discrimination and macrophages of the M1-like type can occasionally exert tumor-promoting functions. A comprehensive correlation between the specific TAM phenotype and function would provide substantial information regarding the role of distinct TAM subsets in BC development/progression. Importantly, TAM spatial and temporal distribution are determining factors for their effects. TAMs located in different breast territories were reported to go through separate differentiation pathways and are characterized by distinct transcriptomic profiles (16). Additionally, TAM phenotype alters with malignancy progression, as tumor stage is one of the key determinants of spatial diversity in tumors (16). Prevalent TAM subsets in BC-based on surface marker expression-are presented next, along with their impact on disease progression and their prognostic value.

### 2.1 CD68 expression

Pan-macrophage marker CD68 was used in initial studies aiming to delineate TAM role in BC progression and prognosis. Increased CD68^+^ TAM infiltration correlated with angiogenesis induction and poor clinical outcomes (17, 18). An association between high CD68^+^ TAM infiltration in the TME and high TNM stage, increased tumor size and shorter patient survival were also reported (19). Distinct functions for TAMs located at different areas in the TME were proposed (20). High stromal CD68^+^ TAM numbers were linked to higher tumor grade, resulting from tubular architecture modulation by TAMs, whereas high numbers of TAMs in the tumor nest were related to angiogenesis. Mahmoud et al. assessed the density and localization of CD68^+^ macrophages in 1322 BC tissues (21). Increased total macrophage numbers were associated with high tumor grade, ER/PR negativity, HER2 positivity and basal BC, while a significant correlation between high macrophage density and reduced BC specific survival was observed. Intratumoral and stromal CD68^+^ TAM infiltration was evaluated in hormone receptor positive and negative BC patient groups (22). High intratumoral infiltration was linked to poor disease-free survival (DFS) in both groups and was an independent DFS predictive factor in the hormone receptor positive group.

### 2.2 CD163/CD206/CD204 expression

CD68 pan-macrophage marker cannot distinguish between macrophages with anti-tumor effects and those with protumor functions. Additional markers were employed to better identify functionally distinct TAM subsets including CD163, CD206 and CD204-scavenger receptors. Increased CD163^+^ macrophage infiltration in tumor stroma positively correlated with higher tumor grade, larger tumor size, Ki67 positivity and ER/PR negativity (23). In the same study, CD68^+^ macrophages in tumor stroma positively correlated with tumor size and were an independent factor for reduced BC specific survival. High stromal CD68^+^ and CD163^+^ TAM infiltration was associated with BC clinicopathological features, increased tumor recurrence and reduced overall survival (OS) (24). Additionally, stromal CD163^+^ macrophages were reported as an independent prognostic factor for relapse-free survival (RFS) and OS. Another study demonstrated that high CD163^+^ TAM numbers were related with increased proliferation and poor differentiation of cancer cells and ER negativity (25). CD163 expression was further linked to negative prognosis and decreased recurrence-free survival. In the same study, conditioned media from the MDA-MB231 breast cancer cell line induced macrophage differentiation into CD163^+^ TAMs *in vitro* via cancer cell secreted CSF-1. The prognostic value of CD68^+^CD163^+^ TAMs was assessed in the tumor nest and stroma of TNBC patients (26). CD163^+^ TAMs in both locations were independent predictive factors for poor prognosis and were associated with reduced OS and RFS. In a separate study, high CD163^+^ TAM infiltration was correlated with increased tumor aggressiveness and reduced progression-free survival (27). The group demonstrated that CD14^+^ blood derived monocytes were converted into CD163^+^ TAMs, upon culture with supernatant from primary dilacerated tumors. Interestingly, distal to the tumor monocytes were refractory to M1 polarization *in vitro* and presented altered transcriptional profile, suggesting a systemic tumor effect. CD68^+^CD163^+^ TAM frequency was studied alongside the frequency of tumor infiltrating lymphocytes (TILs) in TNBC patients (28). High CD68^+^CD163^+^ TAM density, combined with reduced T and B lymphocyte presence significantly correlated with poor prognosis and reduced RFS and OS. Maisel et al., reported that CD163^+^ TAMs in immediate proximity to cancer cells and the average number of CD163^+^ TAMs, either adjacent to or at communicating distance with cancer cells, were independent factors of poor clinical outcomes in BC (29). A separate study in HER2^+^ BC patients revealed a correlation of inferior clinical outcomes with high CD163^+^ TAM density even when HER2-targeted therapy was administered (30). CD163 significance in BC prognosis was highlighted in a recent meta-analysis which used data from 32 studies and identified CD163^+^ TAM density as superior predictor of clinical outcomes compared to CD68^+^ TAM density (31). Strack et al., reported a relative increase to the amounts of CD206^-^ macrophages in BC tumors compared to normal breast tissue (32). Elevated numbers of CD206^-^MHCII^high^ macrophages were correlating with poor prognosis, while CD206^+^ TAMs correlated with improved survival. Similarly, Bobrie et al., reported a positive correlation between CD206 TAM positivity and improved RFS and OS (33). A higher density of CD204^+^ TAMs compared to CD68^+^ or CD163^+^ TAMs was observed in patients with invasive ductal carcinoma (34). High numbers of CD204^+^ TAMs were associated with reduced rates of RFS, distant RFS and BC specific survival. Another study reported CD204^+^ TAM accumulation in highly aggressive breast tumors (35). CD204^+^ TAMs were also prevalent in tumors with increased T lymphocyte infiltration and PDL1 expression and were suggested to contribute to immunotherapy resistance.

### 2.3 PDL1 expression

Increased numbers of CD68^+^/PDL1^+^/CD163^-^ cells at intratumoral sites but not in tumor stroma were associated with favorable clinical outcomes (36). Interestingly, higher CD68^+^/PDL1^+^/CD163^-^ cell density was reported in TNBC and HER2^+^ patients compared to ER/PR^+^ patients. A study in TNBC reported better prognosis in patients with high CD68^+^PDL1^+^ stromal macrophages numbers (37). Superior predictive value for CD68^+^PDL1^+^ macrophages as opposed to PDL1^+^ macrophages was also demonstrated and was proposed as a tool to identify patients with good or poor prognosis. Similar data were obtained by Hong et al. in patients with stage I-III BC, suggesting a positive prognostic role of PDL1 expression on stromal immune cells but not on tumor cells (38). In a single-cell transcriptomic analysis, PDL1^+^ TAMs were reported to be immunostimulatory, demonstrated a preference to localize near T cells and were associated with improved clinical outcomes (39). In another study, increased CD163^+^PDL1^+^ TAM density was associated with advanced stages of BC and metastasis, while PDL1 upregulation was proposed to occur through miRNA mediated gene regulation (40).

The abovementioned studies highlight TAM heterogeneity and underline the necessity for surface marker combinations to accurately identify functionally distinct TAM subsets. A highly specific universal TAM marker for BC prognosis is yet to be discovered. Therefore, a thorough phenotypic characterization of TAM subsets utilizing multiple surface markers remains crucial.

## 3 TAMs orchestrate breast cancer progression and metastasis

Epithelial to mesenchymal transition (EMT) causing loss in cell polarity and cell-cell adhesion, along with destabilization of cell junctions is a driving force of cancer cell migration and invasion. Similarly, stemness induction of cancer cells, endowing them with self-renewal capacities and multi-lineage differentiation capabilities is crucial for metastasis. Extracellular matrix (ECM) remodeling and collagen crosslinking contribute to the metastatic potential by facilitating BC cell migration. TAM involvement in all the above events has been well documented. Recent findings on TAM involvement in BC progression and metastasis are summarized below.

CXCL1, an abundant cytokine in the TME has been associated with poor BC prognosis and increased metastasis (41, 42). TAMs are the main source of CXCL1 in the TME and are involved in EMT induction and tumor cell migration. Their metastatic effect was proposed to occur through the NF-kB/SOX4 axis activation (43). SOX4 implication in EMT induction, cancer stem cell enrichment and poor prognosis in BC patients was also reported by Zhang et al. (44). Induction of CXCL1-secreting M2 TAMs through cancer cell derived visfatin (known adipokine) was reported to promote BC progression and metastasis (45). Increased tumorsphere formation and migration, along with elevated mesenchymal and stemness markers were reported after breast cancer cells were co-cultured with visfatin-treated macrophages. Breast cancer cells from the same co-cultures caused increased pulmonary metastases and high numbers of metastatic nodules in mice, while a CXCL1 blocking antibody reversed those effects. CXCL1 was reported to induce visfatin secretion by cancer cells through a positive feedback loop, thereby maintaining M2 TAM polarization (45).

CCL18 is abundantly expressed by TAMs in BC. TAMs, or myeloid derived monocytes (MDMs) activated with IL-4, promote breast cancer cell invasiveness, adherence to fibronectin and migration *in vitro*, through CCL18 secretion (46). Treatment with an anti-CCL18 antibody, or TAM/MDM transfection with CCL18-siRNAs abrogated cancer cell invasive and migratory capacities. The same group identified a membrane-associated phosphatidylinositol transfer protein 3, PITPNM3 (or Nir1) as CCL18 receptor on cancer cells. In mouse BC xenografts, intratumor rCCL18 injections enhanced vascular invasion of cancer cells and lung and liver metastasis, while breast cancer cell infection with PITPNM3-shRNA alleviated this effect. CCL18 secreting TAMs were reported to be induced by breast cancer cell derived GM-CSF, with lactate —abundant in the TME—acting as a concomitant factor (47). GM-CSF treated TAMs induced cancer cell EMT, migration and invasiveness through NF-kB pathway activation. Importantly, the study demonstrated that both TAM secreted CCL18 and cancer cell secreted GM-CSF are required for the maintenance of cancer cell mesenchymal/metastatic phenotype and macrophage tumor-promoting polarization. Either a CCL18 neutralizing antibody, or an anti-GM-CSF antibody inhibited metastasis in a xenograft mouse model. Annexin A2 (AnxA2)-a member of the calcium dependent phospholipid binding proteins was proposed as a downstream molecule of the CCL18-PITPNM3 signaling in cancer cells (48). A recent study reported the upregulation of exosome derived miR-760 in breast cancer cells stimulated with TAM derived CCL18. This resulted in enhanced cancer cell proliferation and metastatic potential through activation of the ARF6-mediated Src/PI3K/Akt pathway, where ARF6 is a direct miR760 target (49).

IL-1β is a crucial pro-inflammatory cytokine whose aberrant levels were associated with a highly progressive and metastatic potential and poor prognosis in BC patients (50–53). Breast cancer cell lines genetically modified to overexpress IL-1β presented increased EMT and metastasis. In contrast, IL-1β signaling inhibition decreased metastases in a humanized mouse model of BC bone metastasis (51). A recent study suggested a role for IL-1β secreting TAMs in tumor progression and metastasis in TNBC (54). Based on the study, membrane derived soluble CD44 secreted by breast cancer cells triggered IL1-β expression in TAMs promoting cancer cell EMT and metastasis. In mouse models, macrophage ablation or CD44 neutralizing antibody injection, reduced IL-1β serum levels and decreased lung metastasis incidence. CD44 expression on cancer cells was shown to be up-regulated through rhIL-1β treatment, suggesting a positive feedback loop to maintain IL-1β levels. Tsai et al., described the involvement of IL-1β secreting M1 TAMs in BC cell migration and invasiveness (55). BC cell derived GLUT3 triggers lactate-mediated CXCL8 secretion by cancer cells leading to TAM M1 polarization and expression of IL-1β, TNF-α and IL-6. M1 TAMs induced EMT and BC cell migration and invasion through the produced inflammatory cytokines. A paracrine loop between cancer cells and TAMs, whereby TAM derived IL-6 activates STAT3/GLUT3 pathway in cancer cells to preserve high CXCL8 levels was suggested.

A CCL2 paracrine feedback loop between macrophages and cancer cells promotes BC growth and metastasis (56). CCL2 released by cancer cells was shown to increase macrophage migratory capacity and induce M2 polarization *in vitro*. M2 TAM derived CCL2 promoted in turn breast cancer cell stem cell properties. CCL2 expression both in cancer cells and TAMs was shown to be regulated through direct binding of β-catenin to the CCL2 gene promoter. Breast cancer cells overexpressing β-catenin demonstrated high lung metastatic potential and generated larger tumors *in vivo*. Breast cancer growth and breast cancer cell stemness were suppressed through the synergistic effect of CCR2 and β-catenin inhibition. A positive correlation was observed in the expression of β-catenin, CCL2 and CD163 in tissue microarrays from BC patients. Consistent with the above data, CD163^+^ CD206^+^ M2 polarized macrophages were reported to confer stem cell properties and enable EMT of TNBC cell lines through secretion of CCL2 (57). Culture of breast cancer cells in M2 TAM conditioned media enhanced their invasiveness and migratory ability, induced their mesenchymal phenotype and enriched the cancer stem cell population (CD44^+^CD24^low/-^ and ALDH^+^). The study proposed a novel mechanism through which TAM secreted CCL2 activates the PI3K/Akt pathway in cancer cells upon binding to its CCR2 receptor. Subsequent elevated expression and nuclear localization of β-catenin promotes EMT and stemness. Both a β-catenin inhibitor and a CCR2 antagonist were reported to reverse these effects. Interestingly, TAM derived CCL2 has been suggested to induce invasiveness in non-neoplastic epithelial cells (58). In the study, TAM co-culture with non-neoplastic MCF10A breast epithelial cells induced EMT, invasiveness and elevated MMP9 expression in the epithelial cells, through TAM-secreted CCL2. In another study, MMP11-overexpressing TAMs promoted HER2^+^ cell migration, induced monocyte recruitment and enhanced angiogenesis (59). These effects were mediated through CCL2 secretion by TAMs. Cancer cell migration resulted from MMP9 expression upon activation of the CCL2-CCR2/MAPK axis. Of note, MMP11 expression by TAMs can reportedly be stimulated by MMP11-overexpressing cancer cells.

Podoplanin (mucin-type sialoglycoprotein) expressing macrophages (PoEMs) were identified as a metastasis promoting TAM subset in mammary tumors (60). Podoplanin in TAMs was suggested to engage Galectin 8 (GAL8) on lymphatic endothelial cells (LEC), promoting β1-integrin activation and macrophage migration and adhesion to LECs. Upon adhesion, PoEMs were shown to induce lymphangiogenesis. They also enable transendothelial cancer cell migration and are involved in extracellular matrix remodeling through local collagen and MMP production. The same study demonstrated that either the use of anti-β1-integrin blockade or GAL8 inhibition reduced lymphatic cancer spread in mice.

Collagen crosslinking is causative of stromal stiffness and is mediated by two enzyme families, lysyl oxidases (LOX) and lysyl hydroxylases (LH or PLOD). TAM involvement in the induction of stromal stiffness and subsequent metastasis was suggested (61). TAMs in BC TME were proposed to be a source of collagen-crosslinking enzymes leading to extracellular matrix remodeling and stromal stiffness. TAM depletion before tumor invasion could reduce lung metastases in mice, while anti-CSF1 treatment (inhibiting TAM recruitment) decreased stromal LOX and PLOD secretion and reduced the collagen content and number of collagen crosslinks. Stromal PLOD2 expression correlated with poor prognosis in cancer patients.

## 4 Discussion

**Figure 1:**
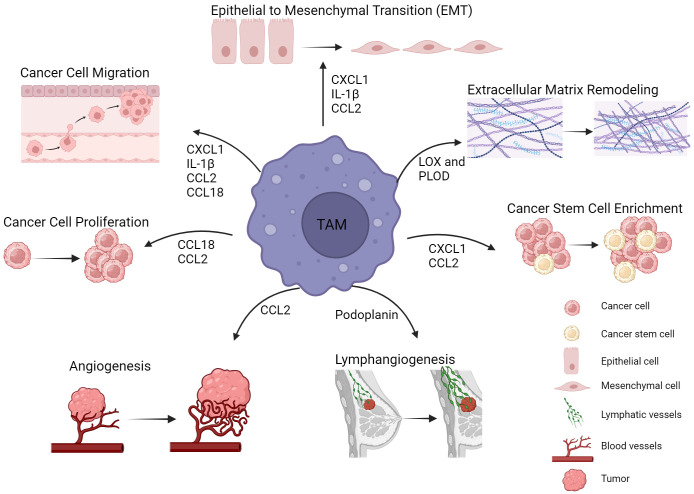
TAMs in breast cancer progression and metastasis. TAMs are involved in multiple mechanisms leading to tumor progression and metastasis. Such tumor promoting mechanisms include epithelial to mesenchymal transition, extracellular matrix remodeling, cancer stem cell enrichment and angiogenesis. Cytokines, enzymes and other factors derived from TAMs are key mediators of these processes. Figure created with BioRender.com.

TAMs are abundant in the TME in BC and their role in promoting disease progression has been well documented. They are involved in multiple aspects of tumor progression and metastasis including cancer cell EMT, stemness induction and extracellular matrix remodeling (**Figure 1**). Importantly, a continuous crosstalk between cancer cells and TAMs is in place to establish and preserve TAM tumor promoting functions and perpetuate cancer cell malignant properties. Given the TAM contribution in BC progression, TAM targeting either as monotherapy, or combined with other therapeutic modalities (chemotherapy/radiotherapy/immunotherapy) poses as a very attractive approach. TAM depletion or blockade of their recruitment to the tumor site, TAM re-education to the tumoricidal M1-like type and enhancement of TAM phagocytotic potential are among the main strategies currently explored (62, 63). Although very promising, these approaches are yet to show their high potential in the clinical setting. This could be at least in part attributed to the considerable heterogeneity of this cell population and the lack of specific and reliable markers to selectively target the desired subsets. Targeting TAMs as a general cell population would entail targeting subsets with anti-tumor effects alongside the tumor-promoting ones. More comprehensive analyses of the different TAM subsets, including analyses at the single-cell level should be considered to enable the identification of highly specific markers to discriminate between functionally different TAM subsets. Additionally, TAM spatial distribution should be accounted for when TAM targeting strategies are designed. TAM functional properties are highly influenced by their specific localization and targeting TAMs at certain sites might offer greater benefit as a treatment approach. It is also worth noting that although TAMs represent primary drivers and facilitators of metastasis, other cells in the TME including cancer associated fibroblasts are also contributing to these processes (64). Therefore, a sole focus on TAMs might not be sufficient to inhibit metastasis and disease progression since other cells could mediate a compensatory effect. Finally, better elucidation of the mechanisms used by TAMs to facilitate disease progression/metastasis along with thorough characterization of the tumor molecular landscape (i.e. expression of high levels of CD44/visfatin/miR760) could provide alternative targeted therapies for individual patients.

## Author contributions

MS: Conceptualization, Writing – original draft, Writing – review & editing. AC: Conceptualization, Supervision, Writing – review & editing.
